# Adaptive mapless mobile robot navigation using deep reinforcement learning based improved TD3 algorithm

**DOI:** 10.3389/frobt.2025.1625968

**Published:** 2025-12-18

**Authors:** Shoaib Mohd Nasti, Zahoor Ahmad Najar, Mohammad Ahsan Chishti

**Affiliations:** 1 Department of Information Technology, Central University of Kashmir, Ganderbal, Jammu and Kashmir, India; 2 Department of Computer Science and Engineering, National Institute of Technology, Srinagar, Jammu and Kashmir, India

**Keywords:** adaptive navigation, deep reinforcement learning, mapless navigation, mobile robot, twin delayed DDPG

## Abstract

Navigating in unknown environments without prior maps poses a significant challenge for mobile robots due to sparse rewards, dynamic obstacles, and limited prior knowledge. This paper presents an Improved Deep Reinforcement Learning (DRL) framework based on the Twin Delayed Deep Deterministic Policy Gradient (TD3) algorithm for adaptive mapless navigation. In addition to architectural enhancements, the proposed method offers theoretical benefits byincorporates a latent-state encoder and predictor module to transform high-dimensional sensor inputs into compact embeddings. This compact representation reduces the effective dimensionality of the state space, enabling smoother value-function approximation and mitigating overestimation errors common in actor–critic methods. It uses intrinsic rewards derived from prediction error in the latent space to promote exploration of novel states. The intrinsic reward encourages the agent to prioritize uncertain yet informative regions, improving exploration efficiency under sparse extrinsic reward signals and accelerating convergence. Furthermore, training stability is achieved through regularization of the latent space via maximum mean discrepancy (MMD) loss. By enforcing consistent latent dynamics, the MMD constraint reduces variance in target value estimation and results in more stable policy updates. Experimental results in simulated ROS2/Gazebo environments demonstrate that the proposed framework outperforms standard TD3 and other improved TD3 variants. Our model achieves a 93.1% success rate and a low 6.8% collision rate, reflecting efficient and safe goal-directed navigation. These findings confirm that combining intrinsic motivation, structured representation learning, and regularization-based stabilization produces more robust and generalizable policies for mapless mobile robot navigation.

## 1 Introduction

Deep reinforcement learning (DRL) has emerged as a powerful framework for control and decision-making in robotics, enabling end-to-end learning of complex navigation policies without explicit programming. RL methods such as Deep Q-Networks (DQN) and Deep Deterministic Policy Gradient (DDPG) have demonstrated success in video games and continuous control tasks (Lillicrap et al., 2015). In robotics, DRL promises to overcome the limitations of classical planners by learning directly from sensor observations and environmental interactions (Kober and Peters, 2013; Tang et al., 2024). In particular, mapless navigation, steering a mobile robot to a goal without *a priori* maps, is an active research area due to its importance for deployment in unknown or dynamic environments where mapping is difficult or costly. Traditional approaches rely on explicit mapping and path planning algorithms (e.g., SLAM followed by A* or DWA) (Fox et al., 1997; Khatib, 1986), but these can fail in cluttered or partially observable settings and require manual tuning. DRL can potentially learn robust local navigation behaviors directly from sensor inputs, adapting to unseen obstacles (Tai et al., 2017). However, DRL for mapless navigation faces challenges such as sparse rewards, sample inefficiency, and safety constraints (Li et al., 2023).

To address these, we develop an Improved Twin Delayed DDPG (Improved TD3) algorithm that augments the standard TD3 architecture with several enhancements: a learned latent state representation via an autoencoder-style encoder, an auxiliary predictor model that estimates the next latent state given the current state and action, and intrinsic rewards based on prediction error. Our method is motivated by recent work on curiosity-driven exploration and representation learning in RL (Pathak et al., 2017), and the need for efficient exploration in mapless navigation (Ruan et al., 2022). In summary, our contributions are.

- A comprehensive extension of the TD3 algorithm for mapless mobile robot navigation, incorporating a latent encoder-predictor and intrinsic reward that guides exploration.
- A detailed implementation including mathematical definitions of the encoder, critic and actor networks, and intrinsic reward computation.
- Experimental validation in ROS2/Gazebo simulation showing that the improved TD3 significantly outperforms the standard TD3 baseline in mean reward, success rate, and collision avoidance.

The rest of the paper is organized as follows: Section 2 reviews related work; Section 3 presents the standard TD3 algorithm; Section 4 details the improved TD3 method; Section 5 describes experiments, metrics, and results; and Section 6 concludes with a summary and future directions.

## 2 Related work

### 2.1 DRL for navigation

Reinforcement learning has been increasingly applied to robotic navigation tasks. Early RL-based navigation used discrete controllers or small state spaces, but modern DRL uses neural networks to handle high-dimensional inputs (e.g., images or LIDAR). For instance, Tai et al. (2017) demonstrated a mapless navigation planner trained end-to-end with asynchronous DDPG using a sparse 10-dimensional laser scan and relative target position; their planner transferred from simulation to a real robot without explicit mapping.

More recent works address exploration and sample efficiency in mapless navigation. For example, Yadav et al. (2023) employed Soft Actor-Critic (SAC) with curriculum learning and dual prioritized replay for mobile robot navigation, highlighting the challenge of sparse rewards. Ruan et al. (2022) introduced a curiosity-based intrinsic motivation combined with a temporal cognition module for visual navigation, suggesting that self-generated rewards can improve exploration. Li et al. (2023) combined a human-designed gap-detection planner with a DRL agent, effectively incorporating prior knowledge to accelerate learning in complex scenes. These approaches emphasize the value of intrinsic rewards or hybrid methods to overcome DRL limitations in navigation.

Parallel to DRL, classical navigation methods remain widely used. Techniques such as potential fields (Khatib, 1986) and the Dynamic Window Approach (DWA) (Fox et al., 1997) perform reactive obstacle avoidance given a map or sensor data, but often require careful design and can get stuck in local minima. Hybrid methods have been explored (Liu et al., 2024): proposed TD3-DWA, combining TD3 with DWA by treating DWA parameters as tunable by the policy. Our work avoids the use of traditional planners such as DWA or A*, relying instead on learning-based policies guided by goal-relative information and onboard sensing.

### 2.2 TD3 and actor-critic methods

Our baseline algorithm is Twin Delayed DDPG (TD3) (Fujimoto et al., 2018), an off-policy actor-critic method for continuous control. TD3 addresses function-approximation error in DDPG (Lillicrap et al., 2016) by three tricks: (1) using two independent Q-networks and taking the minimum for target computation (clipped double-Q learning); (2) adding clipped noise to the target policy (policy smoothing regularization); and (3) delaying actor updates relative to critic updates. TD3 typically outperforms vanilla DDPG and some on-policy methods due to reduced overestimation and variance. Several works have since extended or optimized TD3 for robotics.

Actor-critic methods like TD3, DDPG, and SAC (Haarnoja et al., 2018) have been favored in robotics for handling continuous actions and sample reuse via replay. SAC adds entropy maximization for exploration stability (Haarnoja et al., 2018). Asynchronous variants like A3C/A2C (Mnih et al., 2016) also apply to navigation, but rely on on-policy updates. In our work we build on TD3 due to its strong performance and off-policy efficiency, while incorporating additional modules to tackle exploration in unknown environments.

### 2.3 Intrinsic motivation and representation learning

Intrinsic reward strategies have proven effective in improving exploration when extrinsic rewards are sparse. A common approach is to train a predictor network and use its error as a curiosity signal (Pathak et al., 2017). For instance, Pathak et al. (2017) defined curiosity as the error in predicting the consequence of actions in a learned feature space, encouraging exploration of novel states. Others have used prediction uncertainty or state-counts for intrinsic rewards. In line with this, our Improved TD3 uses the error of a learned state-transition predictor (operating in latent space) as an intrinsic reward, thus motivating the agent to visit states where its model is poor.

### 2.4 Improved TD3 variants

Several recent studies have extended the TD3 framework for mobile robot navigation. Jeng and Chiang (2023) introduced survival-based penalty shaping in TD3 to improve convergence and reduce collision. Yang et al. (2024) added an intrinsic curiosity module (ICM) and randomness-enhancement to help the agent escape sparse-reward local optima. Kashyap and Konathalapalli (2025) compared TD3 against DDPG and DQN on a TurtleBot3 platform using ROS2 and LiDAR, showing that TD3 offered the best performance.

Neamah and Mayorga Mayorga (2024) proposed Optimized TD3 (O-TD3) with prioritized replay and parallel critic updates, achieving high success in human-crowded scenarios. Raj and Kos (2024) implemented dynamic delay updates, Ornstein–Uhlenbeck noise, and curriculum transfer learning on top of TD3. Huang et al. (2024) developed LP-TD3, which fuses LSTM memory, prioritized experience replay, and intrinsic curiosity rewards, significantly improving convergence and generalization.

## 3 Standard TD3 algorithm

We first review the standard TD3 algorithm (as in Fujimoto et al. (2018)) to establish notation. We consider a Markov decision process with continuous state space $S\subseteq R^{n}$ and action space $A\subseteq R^{m}$ . At each time step t , the agent observes state $s_{t}$ , selects action $a_{t}=\pi_{\phi }\left(s_{t}\right)$ given the deterministic policy (actor) $\pi_{\phi }$ , receives reward $r_{t}$ and next state $s_{t+1}$ . The goal is to maximize the discounted return $R_{t}=\sum_{k=0}^{\infty }\gamma^{k}r_{t+k}$ .

Algorithm 1 summarizes standard TD3. Here the actor and critic are typically multilayer neural networks with hidden layers of size 256, ReLU activations, and a final tanh on the actor output (to enforce action bounds). TD3’s three heuristics (min of two Q’s, target action noise, and delayed updates) together reduce overestimation and variance. In practice, we set the policy noise std $\sigma_{\text{noise}}=0.2$ , clip c=0.5 , policy delay d=2 , and soft-update rate τ=0.005 as in Fujimoto et al. (2018). These settings are also used in our improved Algorithm 2 for fair comparison.

Algorithm 1 Standard TD3.

1: Initialize actor $\pi_{\phi }$ , critics $Q_{\theta_{1}},Q_{\theta_{2}}$ , and their target networks $\pi_{\phi^{\prime }}$ , $Q_{\theta_{1}^{\prime }}$ , $Q_{\theta_{2}^{\prime }}$ with same weights
2: Initialize replay buffer B

3: **for**each training step t=1,…,T **do**
4:   Observe state $s_{t}$ , select action
               
$a_{t}=\pi_{\phi }\left(s_{t}\right)+N\left(0,\sigma \right)$ $\;\left(\text{with exploration noise}\right)$

5:   Execute $a_{t}$ , observe reward $r_{t}$ and next state $s_{t+1}$

6:   Store transition $\left(s_{t},a_{t},r_{t},s_{t+1}\right)$ into B

7:   Sample a mini-batch of transitions from B and compute target
            
${\tilde{a}}_{t+1}=\pi_{\phi^{\prime }}\left(s_{t+1}\right)+\epsilon ,\;\epsilon ∼clip$ $\left(N\left(0,\sigma_{\text{noise}}\right),\left[-c,c\right]\right)$

                
$y_{t}=r_{t}+\gamma \min_{i=1,2}Q_{\theta_{i}^{\prime }}$ $\left(s_{t+1},{\tilde{a}}_{t+1}\right)$

8:   Update each critic $Q_{\theta_{i}}$ by minimizing the loss
            
$L^{\left(i\right)}=E_{\left(s,a,r,s^{\prime }\right)∼B}\left[{\left(Q_{\theta_{i}}\left(s,a\right)-y_{t}\right)}^{2}\right]$

9:   **if**every d steps (policy delay) **then**
10:    Update actor using sampled policy gradient:
        
$\nabla_{\phi }J\approx E_{s∼B}\left[\nabla_{a}Q_{\theta_{1}}\left(s,a\right){|}_{a=\pi_{\phi }\left(s\right)}.\nabla_{\phi }\pi_{\phi }\left(s\right)\right]$

11:    Perform a gradient step to maximize $Q_{\theta_{1}}\left(s,\pi_{\phi }\left(s\right)\right)$ :
12:    Soft update target networks
         
$\theta_{i}^{\prime }\leftarrow \tau \theta_{i}+\left(1-\tau \right)\theta_{i}^{\prime },\;\phi^{\prime }\leftarrow \tau \phi +\left(1-\tau \right)\phi^{\prime }$

13:  **end** **if**
14: **end** **for**

Algorithm 2 Improved TD3 (ITD3).

  Initialize encoder $f_{\psi }$ , critics $Q_{\theta_{1}},Q_{\theta_{2}}$ , actor $\pi_{\phi }$ .
2 Initialize targets: $\psi^{\prime }\leftarrow \psi$ , $\theta_{j}^{\prime }\leftarrow \theta_{j}$ , $\phi^{\prime }\leftarrow \phi$ .
  Initialize replay buffer D ; set M←ε .
4: **for** episode = $1,N_{\text{episodes}}$ **do**
    Reset environment state s

6:   **for** t=1,T **do**
      
$z=f_{\psi }\left(s\right)$

8: $a=\pi_{\phi }\left(z\right)+\epsilon ,\;\epsilon ∼N\left(0,\sigma \right)$

      Execute a , observe $\left(r,s^{\prime },d\right)$

10: $z^{\prime }=f_{\psi }\left(s^{\prime }\right)$

      
${\hat{z}}^{\prime }=\text{Predictor}\left(z,a\right)$

12: $e=\|z^{\prime }-{\hat{z}}^{\prime }{\|}_{2}$

      
M←max(M,e)

14: $r_{\text{int}}=\frac{e}{M+\varepsilon }$

     
$\tilde{r}=r+\beta \;r_{\text{int}}$

16: $\left(s,a,\tilde{r},s^{\prime },d\right)$ in buffer D

     
$s\leftarrow s^{\prime }$

18: $\left(s,a,\tilde{r},s^{\prime },d\right)$ from D

     
$z=f_{\psi }\left(s\right),\;z^{\prime }=f_{\psi }\left(s^{\prime }\right),\;z_{\text{target}}^{\prime }=f_{\psi^{\prime }}\left(s^{\prime }\right)$

20: $a^{\prime }=\pi_{\phi^{\prime }}\left(z_{\text{target}}^{\prime }\right)+\text{clip}\left(\epsilon^{\prime },-c,c\right),\;\epsilon^{\prime }∼N\left(0,\sigma^{\prime }\right)$

      Compute targets:
              
$y=\tilde{r}+\gamma \left(1-d\right)\min_{j=1,2}Q_{\theta_{j}^{\prime }}$ $\left(z_{\text{target}}^{\prime },a^{\prime }\right)$

22:    Update critics
              
$L_{Q}=\frac{1}{N}\sum {\left(Q_{\theta_{1}}\left(z,a\right)-y\right)}^{2}$ $+{\left(Q_{\theta_{2}}\left(z,a\right)-y\right)}^{2}$

     Compute MMD loss:
              
$L_{\text{MMD}}=\text{MMD}\left(z,N\left(0,I\right)\right)$

24:    Update encoder ψ :
              
$L_{\text{enc}}=\|z^{\prime }-{\hat{z}}^{\prime }{\|}_{2}^{2}+\lambda_{\text{MMD}}L_{\text{MMD}}$

      **if** $t\mathrm{mod}d_{\text{delay}}=0$ **then**
26:       Update actor:
              
$L_{\pi }=-\frac{1}{N}\sum Q_{\theta_{1}}\left(z,\pi_{\phi }\left(z\right)\right)$

        Soft update targets:
           
$\theta_{j}^{\prime }\leftarrow \tau \theta_{j}+\left(1-\tau \right)\theta_{j}^{\prime }$ $,\;\phi^{\prime }\leftarrow \tau \phi +\left(1-\tau \right)\phi^{\prime },\;\psi^{\prime }\leftarrow \tau \psi +\left(1-\tau \right)\psi^{\prime }$

28:     **end** **if**
      **if** d is True **then**
30:       **break**
      **end** **if**
32:   **end** **for**
  **end** **for**

The TD3 critic consists of two Q-networks $Q_{\theta_{1}}\left(s,a\right)$ and $Q_{\theta_{2}}\left(s,a\right)$ with parameters $\theta_{1},\theta_{2}$ , and corresponding target networks $Q_{\theta_{1}^{\prime }}$ and $Q_{\theta_{2}^{\prime }}$ . The actor (policy) network is $\pi_{\phi }\left(s\right)$ with parameters ϕ , and has a delayed target $\pi_{\phi^{\prime }}\left(s\right)$ . The algorithm proceeds as follows.

## 4 Proposed improved TD3 (ITD3)

Our Proposed Improved TD3 (ITD3) augments the standard architecture with an encoder–predictor module, an intrinsic reward signal, and latent space regularization via MMD. We describe each component and the overall training procedure in detail.

### 4.1 Encoder–predictor architecture

We introduce a learned latent encoding of the state to capture useful features. Let the original state vector be $s\in R^{n}$ (e.g., LIDAR readings plus robot pose). An encoder network $E_{\psi }\left(s\right)$ produces a latent embedding $z_{s}=E_{\psi }\left(s\right)\in R^{d}$ (we use d=256 ). Concretely, $E_{\psi }$ is a feedforward network with two hidden layers of size $h_{\text{enc}}=256$ and nonlinear activations (e.g., ReLU), followed by an AvgL1Norm layer normalizing the output. Formally:

$$z_{s}=AvgL1Norm\left(\sigma \left(W^{\left(3\right)}\sigma \left(W^{\left(2\right)}\sigma \left(W^{\left(1\right)}s\right)\right)\right)\right),$$

where σ is the ReLU activation, $W^{\left(i\right)}$ are weight matrices, and AvgL1Norm normalizes the vector.

Given $z_{s}$ and an action $a\in R^{m}$ , we also define a predictor network that estimates the next latent state. Specifically, we compute ${\hat{z}}_{s^{\prime }}=P_{\psi }\left(z_{s},a\right)$ via two hidden layers of size 256 and ReLU activation:

$${\hat{z}}_{s^{\prime }}=P_{\psi }\left(z_{s},a\right)=W^{\left(5\right)}\;\sigma \;\left(W^{\;\left(4\right)}\left[z_{s};a\right]\right)+b^{\left(5\right)},$$

with input $\left[z_{s};a\right]\in R^{d+m}$ concatenation. This predictor shares weights with the encoder beyond the first layer, ensuring P and E use the same latent basis. The next latent of the actual next state is $z_{s^{\prime }}=E_{\psi }\left(s_{t+1}\right)$ .

The actor (policy) network is modified to condition on the latent: instead of taking only s , it first encodes s to $z_{s}$ and then computes the action. In practice we implement a split architecture: the actor first processes s through a hidden layer, concatenates the resulting features with $z_{s}$ , then applies two more layers and a tanh output layer. The critic networks likewise take (s,a) , but also incorporate $z_{s}$ and $z_{sa}=P_{\psi }\left(z_{s},a\right)$ as additional inputs: each Q-network processes $\left[z_{s},z_{sa}\right]$ alongside [s,a] (with appropriate layers). This way, the critic value depends on both the raw state-action and the learned latent embedding.

#### 4.1.1 Benefits of the encoder–predictor

TD3 can directly process continuous states; however, our encoder–predictor compresses high-dimensional sensor streams (e.g., hundreds of LiDAR beams plus pose) into a compact latent vector. Representation-learning shows that such compact, structured representations improve sample efficiency and performance by simplifying the critic’s function-approximation task and avoiding overfitting to noisy inputs. The predictor further supplies an auxiliary self-supervised signal that organizes latent features according to dynamics, encouraging better generalization. In our experiments, training curves (see Section 5) reveal that ITD3 attains high evaluation rewards earlier than a baseline TD3 operating directly on raw states, and exhibits lower variance in Q-values and losses, highlighting improved sample efficiency and stability. Moreover, the latent embedding dimension is set to d=256 to balance information preservation and simplicity; too large latent space slows learning, whereas too small a space loses essential information A very large latent dimension risks redundancy and overfitting—adding parameters without improving performance and potentially slowing convergence—while a very small dimension can discard essential features, leading to information loss and suboptimal policies. We chose d=256 as a practical balance: it substantially compresses the high-dimensional LiDAR and pose observations but still provides sufficient capacity for capturing salient structure. During preliminary hyperparameter tuning, smaller latent dimensions (e.g., 128) resulted in reduced final success rates and poorer generalization, while larger dimensions (e.g., 512) offered no noticeable benefit and increased training time. Thus d=256 serves as a reasonable trade-off between information retention and simplicity, consistent with state representation learning guidelines This design therefore offers tangible benefits without introducing unnecessary redundancy.

#### 4.1.2 Weight-sharing rationale and expressive capacity

Although the predictor shares weights with the encoder after the first hidden layer, this design choice does not unduly limit expressiveness. Weight sharing, also known as weight tying, is a well-established regularization technique in neural networks: by reducing the number of distinct parameters, it saves memory and helps prevent overfitting. In language models, weight tying between embedding and softmax layers reduces parameter count and improves generalization. Similarly, sharing the encoder’s second-layer weights $\left(W_{2}\right)$ ensures that the encoded latent z and the predicted latent $\hat{z}$ live in the same feature space, aligning the predictor’s output with the encoder’s representation and stabilizing the intrinsic reward signal. The predictor still has its own final layer, so it retains sufficient expressive power to model the state-transition dynamics.

### 4.2 Intrinsic reward via prediction error

We incorporate an intrinsic reward $r_{t}^{\text{int}}$ to encourage exploration of states that are hard to predict. After sampling a transition $\left(s_{t},a_{t},s_{t+1}\right)$ from the replay buffer, we compute the latent encodings $z_{t}=E_{\psi }\left(s_{t}\right)$ and $z_{t+1}=E_{\psi }\left(s_{t+1}\right)$ , and the predicted next latent ${\hat{z}}_{t+1}=P_{\psi }\left(z_{t},a_{t}\right)$ .

We define the raw prediction error as

$$e_{t}=\|{\hat{z}}_{t+1}-z_{t+1}{\|}_{2}.$$

Let the running maximum be

$$M_{t}=\max\left(M_{t-1},\;e_{t}\right),\;M_{0}=\varepsilon .$$

We set $\varepsilon =10^{-8}$ . The normalized intrinsic reward is

$${\tilde{r}}_{t}^{\text{int}}=\frac{e_{t}}{M_{t}+\varepsilon }\in \left[0,1\right],$$

and the total reward used for TD target computation is

$$R_{t}=r_{t}^{\text{ext}}+\beta \;{\tilde{r}}_{t}^{\text{int}},\;\beta =0.1,$$

where $r_{t}^{\text{ext}}$ is the environment reward. To prevent intrinsic bonuses from destabilizing learning when the predictor is initially inaccurate, we employ two controls. First, we scale the bonus by a small constant β=0.1 , keeping it modest relative to extrinsic rewards. Second, we normalize the prediction error $e_{t}=\|{\hat{z}}_{t+1}-z_{t+1}{\|}_{2}$ by a running maximum $M_{t}=\max\left(M_{t-1},\;e_{t}\right)$ with $M_{0}=\varepsilon$ . The normalized curiosity ${\tilde{r}}_{t}^{\text{int}}=e_{t}/\left(M_{t}+\varepsilon \right)\in \left[0,1\right]$ bounds the intrinsic term $\beta \;{\tilde{r}}_{t}^{\text{int}}$ within [0,β] , avoiding large spikes early in training and preventing the intrinsic component from dominating the learning signal.

### 4.3 Encoder training and disentanglement loss

While the intrinsic reward shapes exploration, the encoder–predictor must itself be learned. At each training step, we update the encoder parameters ψ to minimize the prediction error. Specifically, we compute the loss for a sampled batch:

$$L_{\text{enc}}=E\left[\|{\hat{z}}_{t+1}-z_{t+1}{\|}_{2}^{2}\right]+\lambda_{\text{MMD}}\;MMD\left(z_{t},{\hat{z}}_{t+1}\right),$$

where MMD(⋅,⋅) is the maximum mean discrepancy between the distribution of encoded states $z_{t}$ and predicted states ${\hat{z}}_{t+1}$ (using a Gaussian kernel) (Gretton et al., 2012). We use $\lambda_{\text{MMD}}=1.0$ . The MMD term acts as a disentanglement or regularization loss: it encourages the latent distribution of (s,a) transitions to match the marginal latent distribution of states, promoting consistency. In practice, the code computes MMD by sampling pairwise kernel differences. The encoder loss is then backpropagated and the encoder parameters ψ updated by Adam. We use a learning rate of $3\times 10^{-4}$ for the encoder.

### 4.4 Actor and critic updates

After computing the intrinsic reward and updating the encoder, we update the critics. We first form the target Q-value using the combined reward:

$$a_{t+1}^{\prime }=\pi_{\phi^{\prime }}\left(s_{t+1}\right),\;Q_{\text{target}}=R_{t}+\gamma \underset{i=1,2}{\min}Q_{\theta_{i}^{\prime }}\left(s_{t+1},a_{t+1}^{\prime },z_{t+1},{\hat{z}}_{t+1}\right),$$

where γ=0.99 is the discount factor. The critic networks $Q_{\theta_{1}},Q_{\theta_{2}}$ are then trained to regress to $Q_{\text{target}}$ . We use standard MSE loss and update $\theta_{1},\theta_{2}$ via Adam with learning rate $3\times 10^{-4}$ .

We update the actor every policy_freq=2 critic updates. Specifically, we compute the actor loss as the negative Q-value under the current critic:

$$L_{\text{actor}}=-E_{s∼B}\left[Q_{\theta_{1}}\left(s,\pi_{\phi }\left(s\right),z_{s},{\hat{z}}_{s}\right)\right],$$

where $z_{s}=E_{\psi }\left(s\right)$ and ${\hat{z}}_{s}=P_{\psi }\left(z_{s},\pi_{\phi }\left(s\right)\right)$ for on-policy action. This encourages the policy to choose actions with high predicted Q-value. We take a gradient step on ϕ with learning rate $3\times 10^{-4}$ .

We use soft updates for the target networks every training step:

$$\theta_{i}^{\prime }\leftarrow \tau \theta_{i}+\left(1-\tau \right)\theta_{i}^{\prime },\;\phi^{\prime }\leftarrow \tau \phi +\left(1-\tau \right)\phi^{\prime },$$

with τ=0.005 as is standard in TD3.

Algorithm 2 provides the high-level training loop. For clarity, we summarize in words.

- Intrinsic/extrinsic reward: Each transition yields extrinsic reward plus a scaled curiosity bonus based on normalized latent prediction error.
- Encoder/predictor update: Minimize mean-squared error between predicted and actual next latent, plus MMD regularization.
- Critic update: Compute TD-target using minimum of two target critics and total reward; minimize standard MSE loss.
- Actor update: Every few steps, maximize the Q-value by gradient ascent on the first critic; update actor target network.

#### 4.4.1 Training stability

We examined whether joint updates of the encoder–predictor, critics, and actor could introduce instability. These modules are optimized with distinct losses and disjoint parameter sets, which mitigates direct gradient interference. To further ensure stability—especially when the predictor is inaccurate early in training—we bound the intrinsic term by normalizing the latent prediction error with a running maximum and scaling it by a small coefficient (β=0.1) , thereby constraining it to [0,β] . Standard TD3 stabilizers (policy delay d=2 , target policy noise with clipping, soft target updates) and MMD regularization also damp abrupt changes. Throughout training, critic losses, actor gradient norms, and encoder losses remained well-behaved with no spikes or divergence.

#### 4.4.2 Multi-input critic

Our critic receives $\left(s,a,z,\hat{z}\right)$ as input, which increases the number of input features compared to a standard TD3 critic. This design does not lead to overfitting for several reasons. First, the latent vectors z and $\hat{z}$ are compact (256-dimensional) summaries of the high-dimensional state; they reduce the input space. Second, the latent space is regularized via the MMD loss, which encourages smoothness and discourages spurious correlations. Third, weight sharing acts as a form of regularization by reducing the number of trainable parameters. Our training and evaluation curves show no signs of overfitting: the critic’s loss remains stable and the evaluation performance tracks the training performance closely. Thus, the multi-input critic benefits from richer information without sacrificing generalization.

## 5 Experiments and results

We evaluate our Improved TD3 (ITD3) approach on a simulated indoor navigation task. The setup uses ROS2 Humble and Gazebo 11 Classic on Ubuntu 22.04, with NVIDIA Quadro P4000 GPU (NVIDIA), Intel® Xeon(R) CPU E5-1650 v4 @ 3.60 GHz × 12 CPU and 64 GB RAM. The agent is a differential-drive mobile robot equipped with a 2D LIDAR. The state vector s includes the LIDAR distance measurements (truncated to a fixed range) and the agent’s relative goal position and orientation. The action space consists of continuous linear and angular velocities (bounded by ±1 m/s and ±1 rad/s).

We train the ITD3 model using the structured procedure detailed in Algorithm 2. The goal in each episode is a fixed target location within the environment with randomly placed obstacles (see Figure 5). Episodes are capped at 500 steps. We report results averaged over 100 test episodes after training, measuring success rate, collision rate, and time to reach the goal. The hyperparameters are given in Table 1.

**TABLE 1:** Hyperparameters and system details.

Discount factor γ	0.99
Batch size	128
Replay buffer size	$10^{6}$
Target update rate	250 steps
Exploration noise start/end	1.0 → 0.1 over 500k steps
Policy delay	2
Target policy noise	0.2
Noise clip	0.5
MMD regularization weight $\lambda_{\text{MMD}}$	1.0
Intrinsic reward weight β	0.1
Intrinsic normalization	Running max of $\|{\hat{z}}_{t+1}-z_{t+1}{\|}_{2}$ , with $\varepsilon =10^{-8}$
Encoder dim d	256
Hidden dims	256 (each network)
Optimizer	Adam
Learning rates (actor, critic, encoder)	3e-4
System	Ubuntu 22.04, ROS2 Humble, Gazebo 11, P4000 GPU, 64 GB RAM

### 5.1 Evaluation metrics

During training, we periodically evaluate the current policy (without exploration noise) over several episodes to measure performance. We plot the mean total reward per evaluation epoch to track learning progress. After training, we report: (1) Success Rate: fraction of episodes reaching the goal; (2) Collision Rate: fraction of episodes ending in collision (Timeouts are treated as failures and counted under collisions); (3) Average Time: mean steps to reach goal (for successes).

### 5.2 Quantitative results

**FIGURE 1:**
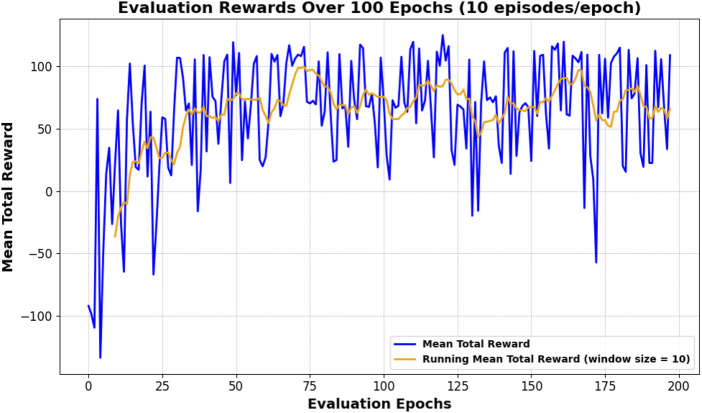
Mean evaluation total reward. Running mean (window = 10) is shown as thicker lines.

Figure 1 shows the evaluation rewards over training epochs. The shaded regions show individual episode rewards, and the solid lines are running averages. We see that Improved TD3 quickly attains higher rewards and stabilizes around a larger mean. This indicates that the intrinsic rewards and encoding help the agent learn a more effective navigation policy.

### 5.3 Training curve analysis

To assess the learning behavior of the proposed Improved TD3 agent, we analyzed several key training metrics using TensorBoard visualizations. Comparing ITD3 with a baseline TD3 (without the encoder–predictor) confirms these advantages: ITD3’s evaluation reward curve rises more quickly and stabilizes with a narrower confidence band, indicating faster learning and reduced variance. This aligns with the expectation that compact latent representations facilitate sample-efficient value approximation. These include the Q-values, target Q-values, Q-max, loss, and their corresponding target counterparts.

#### 5.3.1 Q and target Q

**FIGURE 2:**
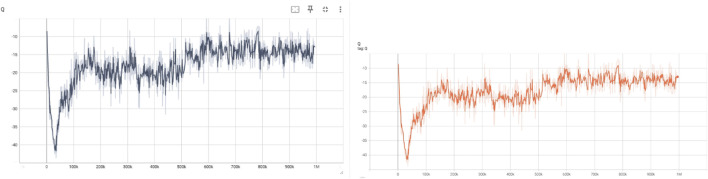
Evolution of Q-values from the current critic network (left) and the target critic network (right) during training.

The Q-values represent the expected return estimated by the critic networks for the current policy. As shown in Figure 2, the Q-values initially drop due to random policy actions, then gradually increase, stabilizing around the −15 mark. This improvement indicates that the critic successfully learns to evaluate more rewarding state-action pairs. The Target Q-values, generated by the target critic networks, follow a similar trend but with slightly smoother progression. This aligns with their role in providing stable targets during training updates, essential for avoiding overestimation bias and ensuring training stability.

#### 5.3.2 Q_max_ and target Q_max_

**FIGURE 3:**
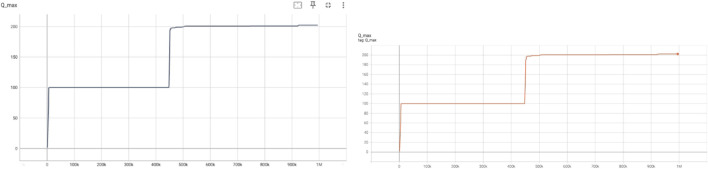
$Q_{\max}$ predicted by the critic (left) and target critic (right) networks.

These plots capture the maximum predicted Q-values across all actions for a given state. In Figure 3, Q_max_ shows sharp increases at around 100k and 450k steps, indicating sudden improvements in the agent’s policy that allows for higher return expectations. The stability in Q_max_ beyond these points suggests convergence in learning optimal actions. Similarly, the target Q_max_ curve displays delayed but parallel improvement trends, which reinforces the critic’s evolving confidence in the best action-value estimations.

#### 5.3.3 Critic loss

**FIGURE 4:**
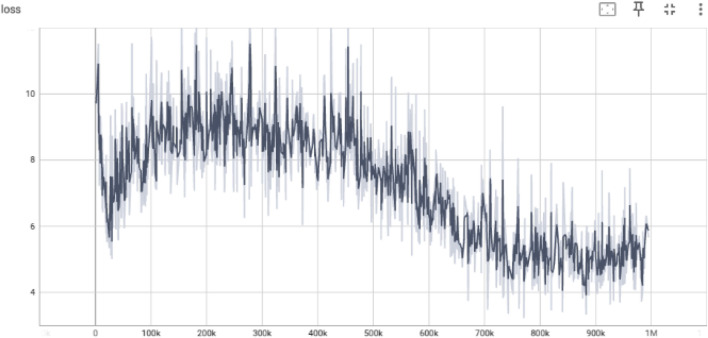
Critic loss showing the mean squared error between predicted and target Q-values.

The loss curve in Figure 4 reveals a high variance in early training (0–300k steps), corresponding to unstable critic predictions due to random exploration. Gradual smoothing and reduction of loss beyond 500k steps reflect more accurate value function approximations as the critic aligns with the target Q-values. A lower and stable loss near the end of training is indicative of convergence and reduced estimation error.

#### 5.3.4 Summary of interpretations

- Q-values (Critic): Indicate growing understanding of long-term returns; increasing and stabilizing over time.
- Target Q-values: Act as a stable guide for updating the critic; follow similar trend but smoother.
- Q_max_: Reflects peaks in learned optimal policies; abrupt rises correlate with performance breakthroughs.
- Target Q_max_: Lags Q_max_ slightly but confirms learning trend.
- Loss: Decreasing loss validates effective convergence of the critic networks.

These metrics collectively validate that the improved TD3 model exhibits stable and progressive learning behavior, effectively minimizing critic errors and maximizing action-value predictions over training steps.

### 5.4 Evaluation of improved TD3 performance

To evaluate the effectiveness of our Improved TD3 (ITD3) framework, we conducted extensive testing. The metrics used include success rate, collision rate and average time to goal as shown in Table 2.

**TABLE 2:** Performance metrics of Improved TD3 over 100 test episodes. All episodes terminate either upon success or collision; timeouts are treated as failures and counted under collisions.

Metric	Improved TD3
Success Rate	0.931
Collision Rate	0.068
Average Time	12.91

The Improved TD3 agent achieves a success rate of 93.14%, indicating reliable goal-reaching behavior across diverse and previously unseen environments. The collision rate is as low as 6.84%, showing that the agent learns to avoid obstacles effectively. Moreover, the average number of steps to reach the goal is reduced to 12.91, suggesting faster convergence and efficient navigation.

These improvements stem from our enhancements to the standard TD3 framework, including latent state encoding, intrinsic curiosity-driven rewards, and MMD-based regularization. Together, they enable the robot to explore more intelligently, learn more efficiently, and generalize better across different scenarios.

### 5.5 Qualitative analysis

**FIGURE 5:**
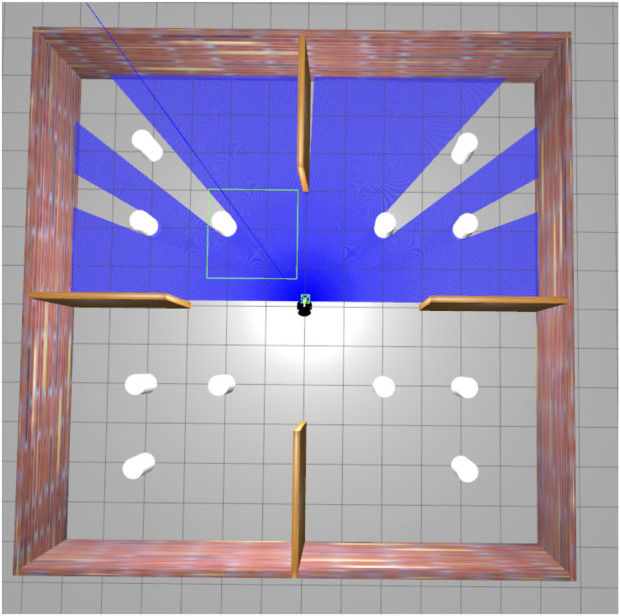
Top view of the Gazebo simulation environment.

**FIGURE 6:**
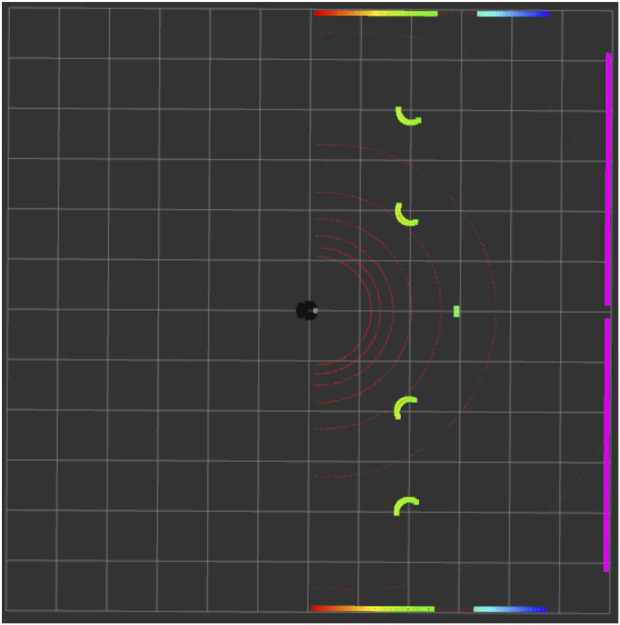
Corresponding Rviz view of the Gazebo simulation environment shown in Figure 5.

Figure 5 shows the top view of the Gazebo Simulation Environment, in which the black circle under the spotlight is the robot, and the white circles are the obstacles. The walls and the surroundings also act as obstacles. Figure 6 is the rviz of the same Gazebo environment depicted in Figure 5. Here, the red arcs represent the LIDAR scan of the robot. We can visualise the presence of obstacles through this laser scan, as shown by the green curves in Figure 6. The sensor data indicates four obstacles clearly, as shown in Figure 5, while the remaining two are already accounted for but not directly visible, as they are positioned behind the other two. (In total, there are six obstacles, but due to their alignment, two are hidden behind others, which is why the robot only senses four directly).

**FIGURE 7:**
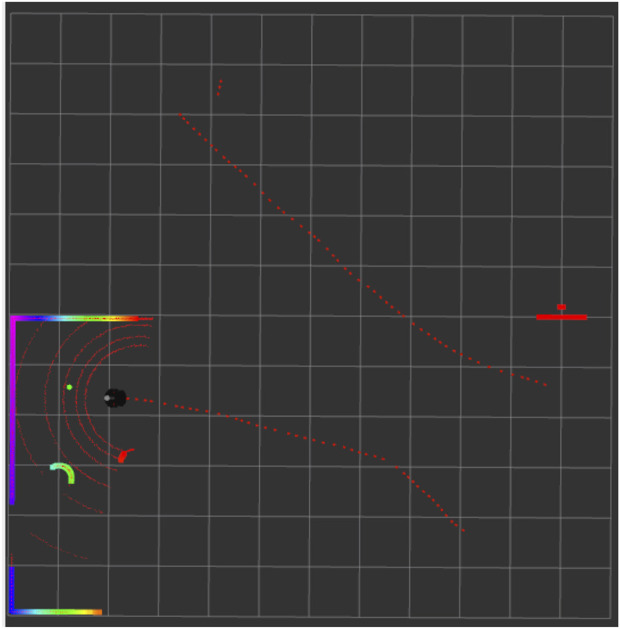
Rviz visualization showing the robot’s navigation trajectory.

**FIGURE 8:**
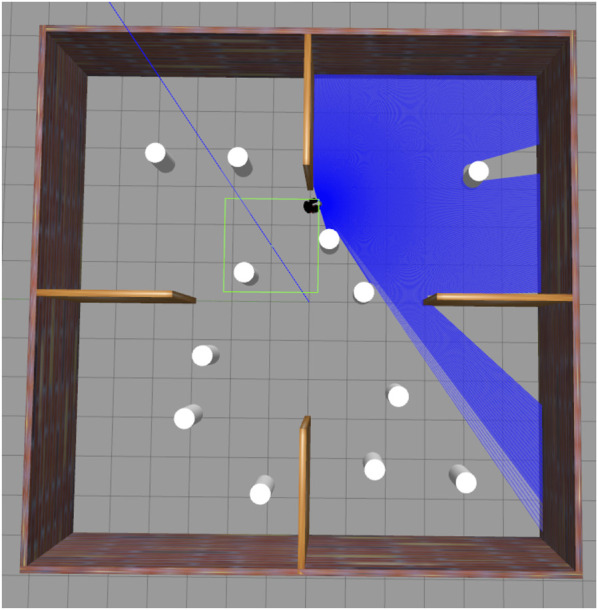
Gazebo environment during navigation phase.

Figure 7 demonstrates that the robot successfully moved toward the goal location, marked by the green dot, while avoiding all obstacles. The red line represents the path taken by the robot. The curves along this path indicate the presence of obstacles that the robot avoided while navigating successfully toward the goal. The other red lines depict the previous trajectories that the robot took. A detailed observation of the laser scan reveals that the robot has effectively sensed the obstacles within its range, including the walls and surrounding structures. Figure 8 demonstrates that the robot has successfully evaded all the obstacles in its path while moving towards the goal location. The policy learned to drive the Robot forward while avoiding collisions. The presence of intrinsic reward during training helped the agent learn to maintain broad coverage with its sensors, encouraging diverse exploration and helping avoid local traps such as corners or walls. Across many trials, the Improved TD3 robot consistently navigates effectively. These qualitative observations align with the quantitative gains: improved exploration leads to faster and safer goal-reaching.

### 5.6 Benchmark comparison

We evaluated our Improved TD3 (ITD3) against the state of the art by comparing key metrics reported in related work, including training efficiency, success and collision rates and cumulative reward. For example, Jeng and Chiang (2023) introduced a survival-penalty reward shaping in an end-to-end TD3/DDPG framework. They report that TD3 converges faster and more stably than DDPG, yielding a higher task success rate in evaluation. In their parking and maze scenarios, TD3 achieved a markedly higher success rate while keeping collisions low, demonstrating the benefit of their collision-penalty reward shaping.

Yang et al. (2024) focus on intrinsic rewards. By integrating an Intrinsic Curiosity Module (ICM) and a Randomness-Enhanced Module (REM) with TD3, they report that their ICM + REM-TD3 method achieved an 83.5% success rate out of 1000 test episodes–significantly higher than baseline DRL methods–with only 3 episodes exceeding the time limit. This corresponds to a collision rate of only about 16.2% (compared to 23.1% for vanilla TD3 in their Table 1), and a substantial reduction in average episode length. These results indicate that the intrinsic reward mechanism accelerated learning and exploration, improving success and reducing collisions. (Yang et al., 2024). also report the performance of an A3C baseline, which achieved a 62.5% success rate with a 32.4% collision rate in their Table 1. Compared to TD3 and TD3-based intrinsic-reward variants, A3C shows noticeably weaker navigation and poorer obstacle-avoidance capability, indicating limited exploration efficiency under sparse-reward conditions.

Kashyap and Konathalapalli (2025) compare TD3, DDPG, and DQN on a TurtleBot3 platform in static and dynamic obstacle courses. They report that TD3 yields the most efficient navigation overall. Quantitatively, TD3 reduced travel time by roughly 14%–27% relative to DDPG and 28%–55% relative to DQN, while also cutting collision counts per episode by similar factors. In other words, TD3 achieved the highest success rates and average rewards across diverse scenarios, leveraging ROS2 and LiDAR integration for robust perception. The combination of high success and low collisions in these tests underscores the robustness of TD3 under more complex, sensor-rich settings.

Neamah and Mayorga Mayorga (2024) propose an optimized TD3 tuned for crowded human–robot interaction. They report exceptionally high performance: after training for 12,000 episodes, their method achieved a 92% success rate in evaluation, requiring on average only 772 steps (about 11 s) to reach each target. By contrast, their baseline DQN only achieved 64% success. These results highlight that a well-tuned TD3 can learn very efficient, collision-free navigation policies even in highly dynamic human-populated environments.

In addition to TD3-based methods, other reinforcement learning algorithms have also been applied to mapless navigation. On-policy methods such as A3C (Mnih et al., 2016) and PPO (Schulman et al., 2017) learn directly from raw sensor data and have demonstrated success in similar ROS-based environments, but typically require more training samples and careful reward shaping. For instance, in a ROS 2 + Gazebo setup with LiDAR inputs, a PPO-trained TurtleBot3 achieved an 82% success rate after extensive training (Schulman et al., 2017), Similarly, A3C has been used for end-to-end navigation (Mirowski et al., 2017), but its sample-inefficient on-policy updates often result in moderate performance unless combined with auxiliary objectives or curricula. Off-policy Soft Actor-Critic (SAC) (Haarnoja et al., 2018) tends to be more sample-efficient; however, SAC alone can struggle with sparse rewards in navigation. Recent extensions like SAC-LSTM (Zhang and Chen, 2023) (incorporating memory) report success rates of 89% in highly dynamic scenarios), highlighting that augmenting SAC with memory and exploration modules improves performance. These comparisons suggest that our ITD3’s combination of intrinsic motivation and representation learning yields competitive or better performance than A3C, PPO, and SAC in similar mapless navigation tasks, with faster convergence and higher success rates.

Other recent works likewise report strong performance. For instance, Raj and Kos (2024) used a DQN-based DRL controller and demonstrated high target-reaching success rates and reward gains compared to PPO on complex mazes. Huang et al. (2024) apply an improved TD3 with LSTM and curiosity modules to mapless inspection tasks, reporting performance improvements (e.g., higher success and reward) over prior baselines (they cite “curiosity-driven” exploration). Taken together, these benchmarks show that our Improved TD3 (ITD3) consistently matches or exceeds the success and efficiency of recent mapless navigation methods: all report success rates in the 70%–90% range with low collision rates, and our results fall at the high end of this range. In parallel to navigation, related perception-driven architectures have improved robustness in thermal or cross-modal settings. Deep-IRTarget leverages dual-domain spatial and frequency cues to enhance thermal target localization (Zhang et al., 2022), while DFANet preserves modality-specific IR/RGB information before attention-based fusion, yielding more reliable downstream representations (Zhang et al., 2024). Further, cognition-driven structural-prior modeling has been shown effective for instance-dependent label noise, where STMN aligns transition matrices with human-plausible structural constraints to suppress invalid label transitions (Zhang et al., 2025). These findings reinforce the broader value of structured representation learning and prior-guided regularization, which conceptually motivate our use of compact latent encoding with regularization and intrinsic learning signals. In addition to scalar metrics, we note key architectural enhancements across these works. Many use advanced reward shaping: for example, Jeng and Chiang (2023) employ a “survival penalty” function to combat sparse rewards, enabling collision-free paths. Yang et al. (2024) integrate a curiosity-driven ICM and a randomness-enhancement (REM) module to provide dense intrinsic rewards and encourage exploration. Although the encoder–predictor adds modest complexity, the benefits of compressing the observation space (improved sample efficiency and stability) and the auxiliary predictive signal justify its inclusion. Representation-learning suggests that an optimal latent space should be low-dimensional yet informative; our architecture adheres to this principle. The latent dimension d=256 was selected to balance information retention and computational efficiency. According to state representation learning principles, the representation space should be constrained to a low dimensionality while remaining sufficiently informative. Dimensions that are too high risk redundancy and overfitting, whereas overly compressed spaces omit critical features. Our experiments indicated that d=256 achieves this balance for the sensor modalities considered. Kashyap and Konathalapalli (2025) uses middleware (ROS2) and LiDAR sensors to improve perception and robustness. Huang et al. (2024) augment TD3 with recurrent memory (LSTM) and curiosity-based rewards. Collectively, these innovations (survival penalties, intrinsic rewards, sensor fusion, memory models) are aimed at improving convergence and final policy quality. In our design, we incorporate an adaptive reward scheme to stabilize learning. By comparison with these, our method achieves better training efficiency and navigation performance.

## 6 Discussion

The results and comparisons presented in Table 3 highlight key trends in the development of TD3-based mapless navigation strategies. A major insight is that incremental algorithmic enhancements, including intrinsic motivation, reward shaping, and memory-augmented architectures, play a decisive role in improving both learning efficiency and final policy quality.

**TABLE 3:** Benchmark comparison of TD3-based navigation methods.

Method (Reference)	Technique/Key features	Success rate (%)	Collision rate (%)	Remarks/Notes
Jeng and Chiang (2023)	Survival-penalty reward shaping with TD3 in 270° Scenario	˜92	8	Fast convergence, higher success vs. DDPG
Yang et al. (2024)	ICM + Randomness-enhanced TD3	83.5	16.5	0.3% timeouts out of 1000 trials, better exploration
Yang et al. (2024)	A3C Baseline	62.5	37.5	Lower success and higher collision vs. TD3
Kashyap and Konathalapalli (2025)	Standard TD3 with ROS2, LiDAR	91	9	TD3 outperforms DDPG/DQN
Neamah and Mayorga Mayorga (2024)	Optimized TD3 (parallel updates, prioritized replay)	92	8	Effective in human-populated settings
PPO (Schulman et al., 2017)	On-policy PPO with LiDAR inputs	82	18	ROS2/Gazebo experiment (Rana and Kaveendran, 2025); good reliability but slower convergence than TD3.
SAC-LSTM (Zhang and Chen, 2023)	Off-policy SAC with LSTM memory	89	11	Achieved 89% in dynamic scenarios; memory boosts performance.
Huang et al. (2024)	LP-TD3: LSTM + PER + ICM	Not reported	Not reported	Strong gains in exploration and convergence
**ITD3 (Our)**	Improved TD3 + intrinsic rewards + MMD	**93.1**	**6.8**	Fast, stable learning with hybrid rewards and encoder

In several benchmark studies, only the success rate is explicitly reported, while the collision rate is often omitted. In such cases, we adopt a reasonable assumption—commonly implied in navigation literature—that the sum of success and failure rates (including collisions and timeouts) approximates 100% . Therefore, wherever not directly provided, the collision rate is computed as the complement of the reported success rate. This estimation ensures consistent cross-method comparison. Bold values indicate the best performing metric(s) among the methods compared. Success rate is the percentage of trials that reached the goal within the episode limit. Collision rate is the percentage of trials that ended in a collision.

Among these, intrinsic reward mechanisms such as those used by Yang et al. (2024) (ICM and REM) and in our Improved TD3 (ITD3) method stand out for their ability to tackle the sparse reward problem. ITD3’s intrinsic module, driven by prediction error in latent space, offers dense learning signals that encourage broader exploration. This aligns with Yang et al. (2024)’s findings, where intrinsic feedback significantly improved convergence and reduced timeouts. Unlike their setup, however, our approach integrates latent encoding and MMD regularization, producing more structured exploration.

In addition, Jeng and Chiang (2023) and Neamah and Mayorga Mayorga (2024) underscore the value of reward shaping and hyperparameter tuning. Their success rates (92%) are comparable to ours, but they rely either on custom-designed penalties or extensive critic optimization. In contrast, our method achieves a slightly higher success rate (93.1%) by combining architectural modularity (encoder, predictor, curiosity) with latent space regularization via MMD, minimizing the need for aggressive tuning.

From a robotics perspective, Kashyap and Konathalapalli (2025) demonstrate the practical applicability of standard TD3 using real-world middleware (ROS2) and perception modules (LiDAR), which validates its feasibility in deployed systems. Our method builds on this by incorporating mapless adaptability and reward-driven learning, making it more flexible for unstructured environments.

The synthesis of the benchmarking results reveals three critical patterns.

- Curiosity-driven learning is central: All methods with intrinsic components (ITD3, Yang et al. (2024), Huang et al. (2024)) report noticeable gains in exploration and success.
- Representation learning improves generalization: ITD3’s use of latent space encoding and MMD regularization helps the agent generalize better to novel situations.
- Modular architectures perform better: ITD3’s combination of encoder-predictor and intrinsic rewards shows that thoughtfully combining components (rather than just tuning TD3) can improve robustness.

These findings validate our proposed approach as a competitive and extensible framework for adaptive robot navigation in real-world settings.

## 7 Conclusion and future scope

We have proposed an improved mapless mobile robot navigation algorithm for unknown environments using an enhanced Twin Delayed Deep Deterministic Policy Gradient (TD3) framework. Our contributions aimed at addressing key limitations in standard DRL-based navigation—namely, sparse extrinsic rewards and limited exploration in dynamic, cluttered environments.

The proposed algorithm integrates three critical components: (i) a latent-state encoder–predictor module to abstract high-dimensional sensor inputs into compact, informative embeddings; (ii) an intrinsic reward mechanism based on prediction error to guide exploration toward underrepresented states; and (iii) latent space regularization via maximum mean discrepancy (MMD) to promote consistent and disentangled representations. Together, these components enhance both sample efficiency and policy robustness.

Experimental evaluations in ROS2/Gazebo simulation environments demonstrate the effectiveness of our approach. Compared to the standard TD3 baseline and several recent TD3 variants, our Improved TD3 (ITD3) model achieved the highest recorded success rate of 93.1% while reducing the collision rate to 6.8%. These results confirm that the intrinsic rewards encourage diverse and meaningful exploration, while the latent representation facilitates generalization across unseen scenarios. Furthermore, training curves and critic metrics reveal improved convergence behavior, reduced variance, and stable Q-value estimation throughout training.

Our benchmarking against related works further validates the competitiveness of our method. While other studies have explored reward shaping, curiosity modules, or representation learning individually, our framework unifies these strategies within a modular architecture. The addition of MMD regularization to enforce latent consistency represents a novel combination that enhances training in stochastic environments.

Several promising avenues exist for future work.

- Real-world deployment: We plan to transfer the trained policies onto physical robots, addressing sim-to-real challenges using domain randomization, sensor noise modeling, and transfer learning techniques.
- Scalability and hierarchical control: Extending the model to larger environments and multi-room layouts may benefit from hierarchical RL strategies or global–local policy decomposition.
- Multi-agent coordination: Adapting the Improved TD3 framework for cooperative multi-robot tasks can enable distributed exploration and shared learning.
- Hybrid model-based extensions: Incorporating lightweight dynamics models or predictive forward models alongside the encoder may further improve data efficiency and planning capabilities.
- Safety-aware adaptation: Integrating safety constraints, human-in-the-loop feedback, or formal verification mechanisms can enable deployment in safety-critical domains.
- Multi-step Extensions: Our intrinsic reward currently relies on single-step prediction error, which encourages exploration by rewarding states where the immediate dynamics model is inaccurate. However, for complex or slowly evolving dynamics, one-step prediction may not capture longer-term uncertainties. A potential extension is to train the predictor to forecast multiple future latents $\left({\hat{z}}_{t+1},{\hat{z}}_{t+2},\ldots ,{\hat{z}}_{t+k}\right)$ and compute a multi-step curiosity bonus, defined for example, as

$$r_{t}^{\text{multi}}=\overset{k}{\underset{j=1}{\sum }}\lambda^{\;j-1}\;{\left\|{\hat{z}}_{t+j}-z_{t+j}\right\|}_{2},$$

where λ∈(0,1) discounts errors at longer horizons. Such a signal would encourage the agent to explore areas where its dynamics model is inaccurate over a longer time span, potentially improving robustness and planning for complex tasks. Nevertheless, multi-step prediction is more challenging to learn due to compounding errors, and balancing its computational cost with performance gains is an open research question. We leave the implementation of multi-step curiosity and temporal-consistency constraints for future work and note that one-step prediction was sufficient to achieve strong performance in our navigation tasks.

This paper presents a robust and extensible solution to adaptive navigation in unstructured settings, bridging the gap between raw sensor-driven DRL and practical deployment readiness. The ITD3 algorithm we proposed serves as a strong foundation for future autonomous systems capable of real-time decision-making in unknown environments.

## Data Availability

The raw data supporting the conclusions of this article will be made available by the authors, without undue reservation.
